# Well-Being and Chronic Disease Incidence: The English Longitudinal Study of Ageing

**DOI:** 10.1097/PSY.0000000000000279

**Published:** 2016-04-11

**Authors:** Judith A. Okely, Catharine R. Gale

**Affiliations:** From the Centre for Cognitive Ageing and Cognitive Epidemiology, Department of Psychology (Okely, Gale), University of Edinburgh, Edinburgh, UK; and MRC Lifecourse Epidemiology Unit (Gale), University of Southampton, Southampton, UK.

**Keywords:** CASP-19, chronic disease, well-being, aging, **BMI** = body mass index, **CVD** = cardiovascular disease, **ELSA** = English Longitudinal Study of Ageing, **SD** = standard deviation

## Abstract

**Background:**

Previous research suggests that greater well-being may protect against onset of chronic disease. However, it is unclear whether this association is similar across different types of disease.

**Method:**

We used Cox proportional hazards regression to examine the prospective relationship between well-being (measured using the CASP-19 quality of life questionnaire) and incidence of arthritis, cancer, stroke, diabetes, myocardial infarction, and chronic lung disease over 8 years. The sample consisted of 8182 participants 50 years or older from the English Longitudinal Study of Ageing.

**Results:**

After adjustments for established risk factors, a standard deviation increase in CASP-19 score was associated with a decrease in arthritis risk (hazard ratio [HR] = 0.89, 95% confidence interval [CI] = 0.83–0.96) and, in those younger than 65 years, a decrease in diabetes risk (HR = 0.82, 95% CI = 0.70–0.95) and chronic lung disease risk (HR = 0.80, 95% CI = 0.66–0.97). Higher CASP-19 scores were associated with reduced risk for stroke and myocardial infarction; however, these associations were no longer significant after adjustments for established risk factors. No association was observed for cancer incidence. An age interaction was observed for diabetes, myocardial infarction, and chronic lung disease, with a stronger association between CASP-19 score and disease incidence at younger ages.

**Conclusions:**

The extent of association between well-being and incident disease risk is not consistent across different chronic diseases. Future studies should examine the cause of this variation.

## Abstract 2

Supplemental digital content is available in the text.

## INTRODUCTION

The relationship between emotion and physical health has inspired an extensive body of research (1). Numerous studies have documented the link between negative emotions and subsequent health outcomes including cardiovascular disease, disability, and mortality (2–4). More recently, the popularity of positive psychology and focus on well-being in areas of public policy (5) have prompted interest in a less researched aspect of the relation between emotion and health, namely, the relationship between subjective well-being and health outcomes.

Most research in the field of subjective well-being and health has been informed by two distinct perspectives (6). Hedonic well-being refers to the experience of positive emotion such as pleasure or happiness. Eudaimonic well-being refers to longer-term functioning such as self-realization or sense of autonomy.

There is compelling evidence that both hedonic and eudaimonic well-being are positively associated with longevity. In a systematic review of studies into the relation between hedonic well-being and longevity, Chida and Steptoe (7) found that positive affect and positive trait-like dispositions predicted longevity after controlling for negative affect, demographic, and health behavior variables. A similar pattern has been reported regarding the association between eudaimonic well-being and longevity (8,9).

These findings have inspired investigation of the association between well-being and incidence of chronic disease. Greater well-being has been linked with lower incidence of cancer, and breast cancer specifically (10,11), Type 2 diabetes (10,12), and cardiovascular disease (13,14). However, the evidence is inconsistent: other studies have reported no relation between well-being and breast cancer (15) or heart disease (10,16). This line of investigation has raised the question of whether the association between well-being and disease risk is similar across different diseases. Many chronic diseases share several risk factors, but it is unclear whether chronic diseases share another risk factor in the form of well-being. Richman et al. (17) have argued that well-being may provide a “broad base of resilience” against chronic disease. However, Diener and Chan (18) suggested that as different types of disease involve different physiological processes and causes, the strength of association between well-being and disease risk is likely to vary across different types of diseases—with some having little or no association with prior well-being.

Research investigating the association between well-being and multiple disease outcomes is sparse. One study investigating the association between positive emotion and respiratory tract infection, diabetes, and hypertension found a significant association in the case of incident hypertension but not for incident diabetes or respiratory tract infection (after adjusting for established risk factors) (17). However, it is unclear whether well-being was differentially related to these disease outcomes because the study was significantly underpowered. Another, larger study (10) found no significant relationship between well-being and myocardial infarction but greater well-being was associated with a reduced risk of cancer, diabetes, and stroke. In the current study, we aimed to extend this area of research by comparing the association between well-being and disease risk across a greater number of chronic diseases. We used data from the English Longitudinal Study of Ageing (ELSA), a population-based sample of men and women 50 years and older. The sample includes data on the incidence of a range of chronic diseases. Disease outcomes included in the current study were stroke, myocardial infarction, diabetes, arthritis, chronic lung disease, and cancer.

## METHODS

### Study Population

The ELSA is a prospective study of people 50 years and older. Participants were initially recruited from the Health Survey for England database in 1998, 1999, and 2001. At Wave 1 (2002–2003), 12,099 people participated; since then, participants have been interviewed biennially. Ethical approval was provided by the London Multicentre Research and Ethics Committee. Participants gave written informed consent (19).

### Well-Being and Chronic Disease Incidence

Well-being at Wave 1 was assessed with the CASP-19 quality of life questionnaire that assesses the domains of control, autonomy, self-realization, and pleasure (20). Participants respond to 19 questions on a 4-point Likert scale (scored 0-3). Possible total scores range from 0 to 57, with higher scores indicating higher levels of well-being. For individuals with 4 or fewer CASP-19 items missing, we imputed a score for the missing items based on their mean score for the completed items. For the study sample, internal consistency for CASP-19 scores was high (α = 0.87).

At Wave 1, participants were asked whether a doctor had ever told them that they had any of the following conditions: high blood pressure/hypertension, myocardial infarction, diabetes or high blood sugar, a stroke, chronic lung disease, arthritis or rheumatism, or cancer. Participants reported the month and year of diagnosis. In subsequent waves (2–5), participants were presented with the same list and asked whether they had been diagnosed as having any of the listed conditions since their last interview, and if so, what date this occurred. Data regarding time of diagnosis were not available for chronic lung disease. Instead, time of diagnosis was indexed as the time of the interview (month and year) at which the participant first reported a diagnosis of chronic lung disease.

### Covariates

We chose age, sex, depressive symptoms, socioeconomic status, education, and relationship status as potential confounders and health behaviors (physical activity, alcohol consumption, and smoking status), and body mass index (BMI) as mediators of the relationship between well-being and later disease incidence. Feller et al. (10) found that adjusting for health behaviors, BMI, and education attenuated the association between life satisfaction and disease incidence. Socioeconomic status, depressive symptoms, and age have been associated with well-being as well as incidence of chronic disease (5,21–26).

The eight-item version of Centre for Epidemiological Studies Depression Scale (CES-D) was used to assess symptoms of depression (27). Socioeconomic status was indexed by total household wealth; household wealth has been identified as the most accurate indicator of long-term socioeconomic circumstances in ELSA (28). Education was based on highest reported level of qualification: less than O level or equivalent, O level or equivalent, A level or equivalent, higher than A level but below degree, and degree level (American equivalent qualifications are high school diploma for O level and 1-year study at college with a B average for A level). Relationship status was dichotomized as having a partner (yes or no). Participants reported the frequency with which they engaged in vigorous, moderate, and mild exercise. Response options were “more than once a week,” “once a week,” “one to three times a month,” and “hardly ever or never.” As previously (29), responses were dichotomized—either once a week (or more) or less than once a week—and then summed to create four categories: physical inactivity, mild but not moderate/vigorous activity, moderate but not vigorous physical activity, and vigorous physical activity. Frequency of alcohol consumption was recorded. Response options were as follows: twice a day or more, daily or almost daily, once or twice a week, once or twice a month, special occasions only, and not at all. Participants reported their smoking status.

### Analytical Sample

Of the 12,099 people taking part in Wave 1, 8182 were included in the current analysis. Participants were excluded if they only took part in Wave 1 (*n* = 2121; 18%); they were additionally excluded if they had incomplete (missing more than four items) or missing data for well-being (*n* = 886; 7%) and further excluded if they had missing data on any of the covariates (*n* = 910; 8%). The median length of follow-up was 6 years; the last year of assessment considered in this study was 2011.

### Statistical Analysis

Cox proportional hazards regression was used to examine the association between CASP-19 scores at baseline and incidence of diabetes, myocardial infarction, stroke, cancer, chronic lung disease, and arthritis. On the basis of Schoenfeld residuals, we found no evidence that the proportional hazards assumption was violated (all *p* values > .2). Survival time in days was calculated from the date of the Wave 1 interview to the date of diagnosis with the disease of interest or the date of the last follow-up interview, whichever occurred first. Participants diagnosed as having the disease of interest at Wave 1 were excluded from the analysis. Four models were used, in which we first adjusted for potential confounding factors (Models 1-3) and then in addition for potential mediating factors (Model 4). Model 1 adjusted for age and sex. Model 2 additionally adjusted for total household wealth, relationship status, and education, and where appropriate, for comorbidities present at Wave 1, such that for the outcome of diabetes, Model 2 adjusted for reported hypertension; for myocardial infarction and stroke, Model 2 adjusted for reported diabetes and hypertension; and for chronic lung disease, Model 2 adjusted for reported asthma. Model 3 additionally adjusted for CES-D score. Model 4 additionally adjusted for health behaviors and BMI. Hazard ratios (HRs) and 95% confidence intervals (CIs) for each chronic disease are expressed according to a standard deviation (SD) increase in CASP-19 score. To test for age or sex differences in the association between CASP-19 score and disease risk, we entered multiplicative interaction terms (e.g., CASP-19 by age) in an age- and sex-adjusted model. To reduce the risk of reverse causality (i.e., undiagnosed preexisting disease influencing well-being), for outcomes that remained significantly associated with CASP-19 scores in the fully adjusted model, the regression was repeated excluding the first 2 years of follow-up.

We carried out additional Cox regression analyses of incident disease in which we treated well-being and appropriate covariates (health behaviors, depressive symptoms, relationship status, and BMI) as time varying, using variables derived from data at Waves 1 to 4, to account for possible changes in well-being and covariates over follow-up.

## RESULTS

Table 1 shows the baseline characteristics of the sample according to tertiles of well-being. On average, people with higher well-being were younger, had lower CES-D scores, were wealthier, had lower BMI, were more physically active, consumed more alcohol, and had a higher level of education. People with higher well-being were also more likely to be female, in a relationship, not to smoke, and less likely to report a history of arthritis, diabetes, myocardial infarction, hypertension, chronic lung disease, and stroke.

**TABLE 1:**
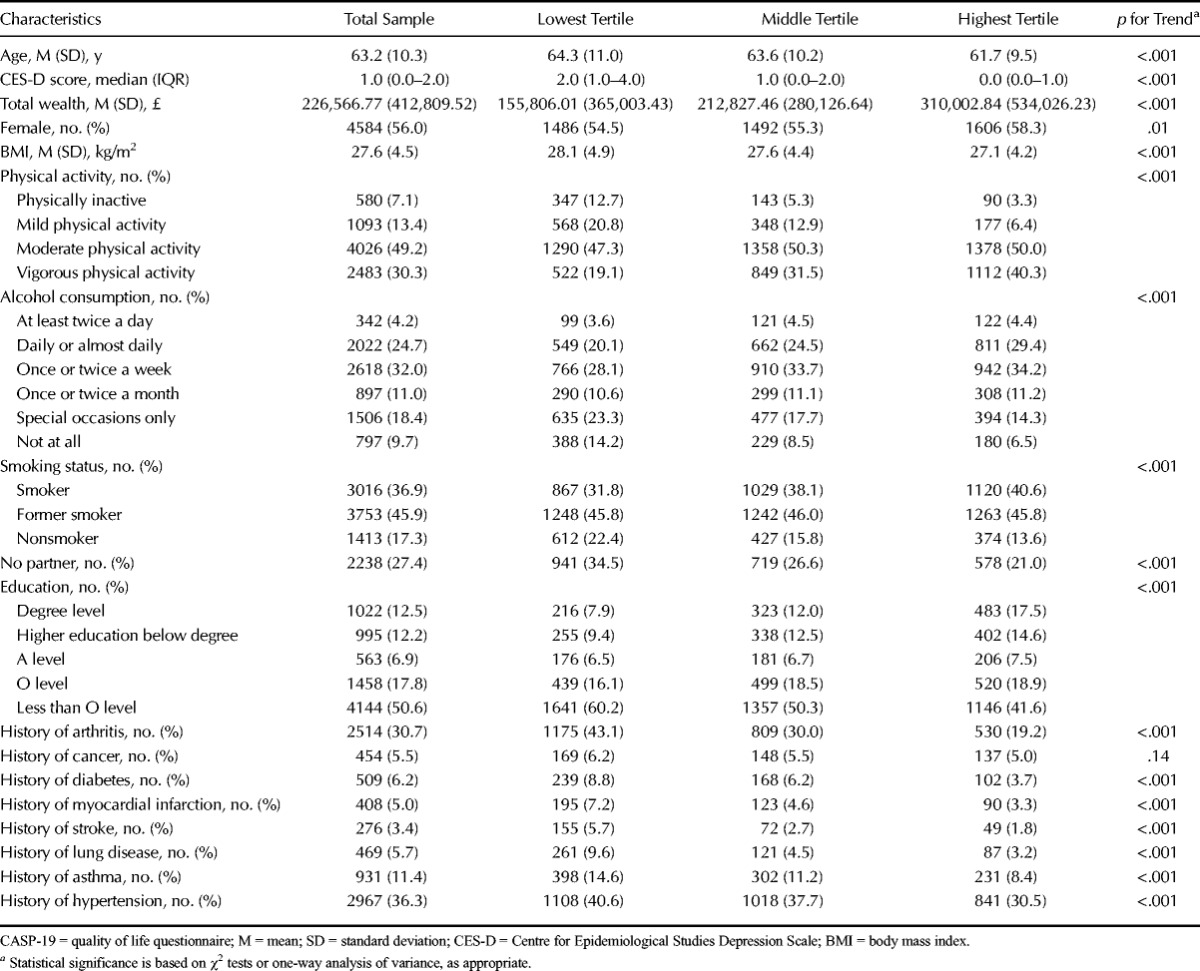
Baseline Characteristics Stratified According to Tertiles of CASP-19 Scores (Lowest, Middle, and Highest Subjective Well-Being); Total *n* = 8182

Preliminary analysis indicated that the relationship between CASP-19 scores and chronic disease incidence did not differ according to sex (*p* for interaction terms all >.05). The relationship between CASP-19 and incidence of diabetes, myocardial infarction, and chronic lung disease did differ significantly by age. In the case of these three disease outcomes, separate Cox proportional hazards regression analysis was conducted for two age groups (<65 and ≥65 years).

Estimates from models that used time-varying measures of well-being and the covariates were very similar to those obtained using the measure of well-being at one time point (Wave 1) only, so only results from these latter analyses are presented here (see Table S1, Supplemental Digital Content 1, http://links.lww.com/PSYMED/A261, for a comparison of results).

Table 2 displays the HRs for incident arthritis, cancer, stroke, diabetes, myocardial infarction, and chronic lung disease according to an SD increase in overall CASP-19 score.

**TABLE 2:**
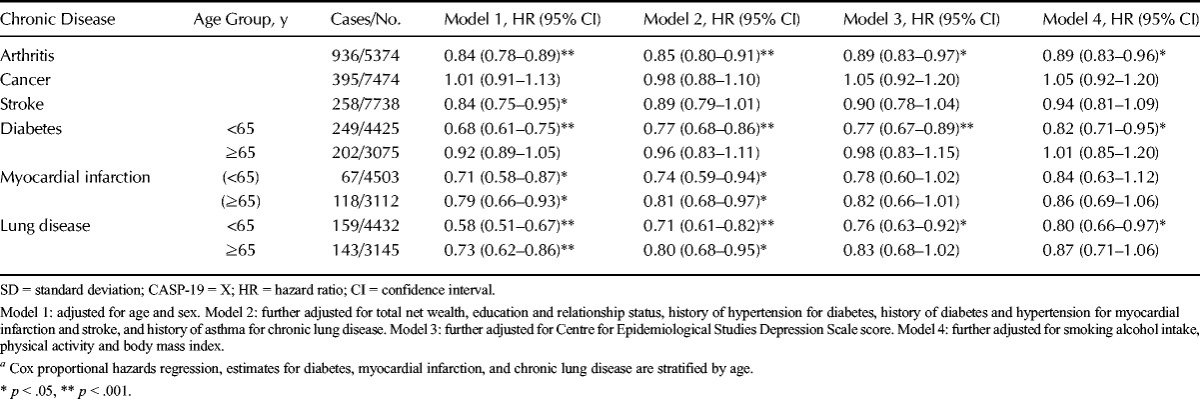
Hazard Ratios (95% Confidence Intervals) for Incident Arthritis, Cancer, Stroke, Diabetes, Myocardial Infarction, and Chronic Lung Disease According to an SD Increase in CASP-19 Scores^*a*^

Nine hundred thirty-six incident cases of arthritis were reported during follow-up. There was a significant inverse association between CASP-19 scores and arthritis risk. This relationship remained significant (although attenuated) after adjusting for potential confounding variables (wealth, education, relationship status, and CES-D score). Adjusting for potential mediating variables (health behaviors and BMI) did not attenuate this association further. The HR in the fully adjusted model was 0.89 (95% CI = 0.83–0.96).

Three hundred ninety-five new cases of cancer were reported. No significant association was found between CASP-19 scores and incidence of cancer.

Two hundred fifty-eight new cases of stroke were reported. A significant association between CASP-19 scores and incidence of stroke was only observed in Model 1 (adjusted for age and sex) with an SD increase in CASP-19 score associated with an HR of 0.84 (95% CI = 0.75–0.95). This association was of borderline significance (*p* = .08) after adjustments for the potential confounding variables in Model 2. Further adjustment for depressive symptoms and the potential mediating variables attenuated the association still further.

Four hundred fifty-one cases of incident diabetes were reported during follow-up. Analysis stratified by age group indicated a trend for a stronger association between CASP-19 scores and incident diabetes at a younger age. In the <65-year age group, a significant inverse association between CASP-19 score and diabetes risk was observed in all four models. The association was attenuated by adjusting for potential confounding variables but remained statistically significant (HR = 0.77, 95% CI = 0.67–0.89). Further adjustment for the potential mediating factors suggested that these variables explained 21% of the association. No significant association was observed for the ≥65-year age group.

One hundred eighty-five new cases of myocardial infarction were reported. There was a trend for a stronger association between CASP-19 scores and incident myocardial infarction at a younger age. A significant inverse association between CASP-19 scores and myocardial infarction risk was observed for both age groups. In Model 2, an SD increase in CASP-19 score was associated with a decrease in myocardial infarction risk (HR = 0.74, 95% CI = 0.59–0.94) for the <65-year age group and a decrease in myocardial infarction risk in the ≥65-year age group (HR = 0.81, 95% CI = 0.68–0.97). This association was of borderline significance after adjusting for depressive symptoms in Model 3 for the <65-year age group (*p* = .07) and the ≥65-year age group (*p* = .06). The association was further attenuated by adjusting for potential mediating variables in Model 4.

Three hundred two new cases of chronic lung disease were reported. There was a trend for a stronger association between CASP-19 scores and incident lung disease at younger ages. In those younger than 65 years, a significant inverse association between CASP-19 scores and chronic lung disease risk was observed in all four models. In Model 3, an SD increase in CASP-19 score was associated with an HR of 0.76 (95% CI = 0.63–0.92). Adjusting for potential mediating variables in Model 4 attenuated the association by 16%. In Model 2, for the ≥65-year age group, an SD increase in CASP-19 score was associated with an HR of 0.80 (95% CI = 0.68–0.95). The association was of borderline significance in Model 3 (*p* = .06) and was further attenuated by adjusting for health behaviors and BMI in Model 4.

We carried out several sensitivity analyses. We tested whether exclusion of participants with missing covariate data had biased the results. For each disease outcome, the age- and sex-adjusted model was rerun including participants with missing covariate data. The results were very similar to those obtained in the sample with complete data indicating that the exclusion of participants with missing covariate data did not significantly affect the results. We also tested for bias due to the exclusion of participants with missing CASP-19 data. In an age- and sex-adjusted model, missing well-being data did not predict incident arthritis, stroke, diabetes, or myocardial infarction; however, participants with missing well-being data were significantly more likely to develop chronic lung disease and were less likely to be diagnosed as having cancer. We carried out additional sensitivity analyses for the three chronic conditions that were significantly associated with well-being in the fully adjusted model: arthritis, diabetes (for those <65 years of age) and chronic lung disease (for those <65 years of age). Firstly, we repeated the analysis excluding cases diagnosed in the first 2 years of follow-up. The association between well-being and risk of arthritis and diabetes was slightly changed suggesting that the association is unlikely to reflect reverse causation whereby undiagnosed disease at baseline affected well-being. However, the association between well-being and chronic lung disease incidence was no longer significant. The fully adjusted HRs for arthritis, diabetes, and chronic lung disease were 0.87 (95% CI = 0.79–0.96), 0.84 (95% CI = 0.70–1.00), and 0.85 (95% CI = 0.67–1.08), respectively. Three hundred fifty-three cases of incident arthritis, 62 cases of diabetes, and 53 cases of chronic lung disease were excluded from this sensitivity analysis. Second, we repeated the analysis using an amended CASP-19 score that excluded two items that refer either specifically to health (“my health stops me from doing the things I want to do”) or to age-related problems that might be interpreted by the respondent to include health (“my age prevents me from doing the things I would like to do") to check whether they inflated the associations we found between well-being and disease risk. When both items were excluded from the CASP-19 score, fully adjusted HRs remained significant for the outcomes of arthritis and chronic lung disease (0.92 [95% CI = 0.85–0.99] and 0.82 [95% CI = 0.68–0.99], respectively) and were of borderline significance in the case of diabetes (HR = 0.86 [95% CI = 0.74–1.00]). These estimates are very similar to those obtained using the original CASP-19 score, although the HR for diabetes is slightly weaker.

## DISCUSSION

In this study of people 50 years and older, those with greater well-being at baseline had a lower risk of incident disease. However, the extent of this association differed according to type of disease and, in some cases, age group. Incident arthritis, diabetes, and chronic lung disease were associated with CASP-19 scores after adjusting for confounding variables; in the case of diabetes and chronic lung disease, the association was partially mediated by health behaviors and BMI. The risk association for myocardial infarction remained significant after adjusting for demographic variables and comorbidity but became of borderline significance after adjusting for depressive symptoms and was no longer significant after adjusting for health behaviors and BMI. For the outcome of stroke, this association became nonsignificant after adjusting for demographic variables, comorbidity, and depressive symptoms. No association was observed for cancer incidence. An age interaction was observed for diabetes, myocardial infarction, and chronic lung disease, with a stronger association between CASP-19 score and disease incidence at younger ages.

This is the first study to report an association between well-being and risk of incident arthritis. The relationship between psychological factors and the onset of arthritis has received little empirical attention (30). However, one longitudinal study has reported a positive association between perceived stress and onset of arthritis 3 years later (31). Examination of the four analytical models suggests that the association between well-being and arthritis risk is partially confounded by demographic variables and depressive symptoms. The association was not explained by the potential mediating factors, health behaviors, and BMI. This suggests that additional mechanisms (not controlled for in our model) are involved in the association between well-being and arthritis risk. Well-being may have an effect on physiological processes relevant to arthritis risk or symptom expression. Higher well-being is associated with a reduced inflammatory response (32,33). Thus, well-being might lessen the expression of arthritic symptoms by reducing the extent of inflammation.

In the present study, we found no significant association between CASP-19 scores and incident cancer. Our results are in line with two studies that reported no association between life satisfaction and breast cancer (15) and life satisfaction and cancer as a whole in men (10). However, our findings contradict reports of a significant association between well-being and cancer in women (10) and breast cancer specifically (11). It is possible that well-being is associated with particular types of cancer. It has been suggested that psychological factors such as stress and depression specifically increase the risk of virus-related cancers (29,34). We were unable to discriminate between different types of cancers in our analysis, and thus may have missed these specific associations.

Higher CASP-19 scores were associated with a decreased risk of incident stroke in the age- and sex-adjusted model. This association did not persist after adjustment for demographic variables and prevalent hypertension and diabetes. Some studies have reported a similar attenuation after adjusting for established risk factors in the case of cardiovascular disease incidence (35,36) and stroke (10). However, others have reported a significant association between positive affect and incident stroke after adjusting for demographic factors, prevalent diabetes, hypertension, and health behaviors (37). Difference in sample age (all >65 years) and the inclusion of fatal stroke cases could explain why this latter study found a stronger association than that found here.

We found a significant association between CASP-19 scores and myocardial infarction risk after adjusting for demographic variables in both age groups. The association was of borderline significance after adjusting for depressive symptoms and further attenuated after adjusting for mediating variables. Two studies have reported a significant association between well-being and incident coronary heart disease after adjusting for established risk factors (14,38); neither study adjusted for the potential mediating effect of physical activity. Feller et al. (10) found no significant association between life satisfaction and incident myocardial infarction. The authors suggest that this null finding may be due to a low number of cases in their sample. The current results support findings indicating an association between well-being and myocardial infarction risk (14,38); however, our results suggest that this association is predominantly confounded by depressive symptoms and mediated by health behaviors and BMI.

Diabetes risk was significantly associated with CASP-19 score for the <65-year age group. This association remained significant but attenuated in the fully adjusted model. In the ≥65-year age group, there was no association between CASP-19 score and diabetes risk. A previous study has documented an association between well-being (emotional vitality and life satisfaction) and physician-diagnosed diabetes (39). In line with our results, this association remained significant after adjusting for demographic variables, health behaviors, BMI, and depressive symptoms. However, a second study into the association between life satisfaction and diabetes risk has documented a weaker association (10). Life satisfaction was only associated with diabetes risk in women, and this association was not significant after excluding incident diabetes in the first 2 years and adjusting for established risk factors and prevalent hypertension. Other psychological factors (not controlled for in the current study) including stress, anger, hostility, sleep problems, and anxiety have been associated with incidence of diabetes. Feller et al. (10) suggest that well-being may provide a buffer against the influence of these psychosocial factors. Alternatively, well-being may directly affect physiological processes relevant to diabetes risk. For instance, elevated C-reactive protein previously associated with low well-being (40) is a strong independent predictor of Type 2 diabetes (41).

For people younger than 65 years, higher well-being significantly reduced the risk of chronic lung disease; this effect remained significant but attenuated after adjusting for established risk factors. In the ≥65-year age group, this association was no longer significant after adjusting for depressive symptoms. This is the first study to document a significant association between well-being and incidence of chronic lung disease. Our results suggest that this association is partially confounded by demographic variables, history of asthma, and depressive symptoms and mediated by health behaviors. However, additional factors appear to play a role in the <65-year age group. Chronic inflammation is central to the pathophysiology of chronic lung diseases (42); thus, similarly to arthritis, well-being may affect chronic lung disease risk via its association with the inflammatory response (32,40).

Our findings suggest that the strength of the association between well-being and disease risk is disease dependent—with no association found for cancer and the strongest associations observed for arthritis, diabetes, and chronic lung disease. It is unclear why this group of diseases has a stronger association with well-being; however, the role of reverse causality should be considered. Compared with the events of stroke and myocardial infarction, clinical diagnosis of arthritis, diabetes, and chronic lung disease may occur sometime after disease onset. The stronger association between well-being and this group of diseases found in our study may perhaps reflect the impact of undiagnosed symptoms at baseline on well-being. Although our results support the idea that well-being is differentially related to different diseases, no previous studies have documented this pattern of specificity. Feller et al. (10), who included a number of the same disease outcomes as the current study (diabetes, all-cause cancer, stroke, and myocardial infarction), report a different pattern of association (with the strongest association between life satisfaction and disease risk in women observed for cancer and stroke). Research involving a comparable age group and well-being measure is needed to confirm our findings.

The time-varying model produced similar estimates to the original model (which used baseline measures of covariates) for the association between overall CASP-19 scores and disease risk. The small difference between the two models may indicate that levels of well-being remained stable over time. The temporal stability of well-being has been documented in a number of studies (43,44). In the current sample, the test–retest correlation coefficient for CASP-19 scores at Wave 1 and Wave 5 was high: *r* = 0.62, *p* < .001.

In the case of diabetes, myocardial infarction, and chronic lung disease, we found that the association between well-being and disease incidence tended to be stronger at younger ages. A similar age interaction—with a stronger effect at younger ages—is reported by a study into the association between life satisfaction and mortality (9). One explanation for our observations is that individuals who were particularly susceptible to the potentially detrimental effects of low well-being on health did not survive to older ages. In contrast with our results, two systematic reviews indicated that the associations between well-being and mortality or cardiovascular and physiological reactivity may, in fact, be stronger at older ages (7,45). The cause of these divergent findings is unclear; however, it is possible that the effect of age is dependent on health outcome. Howell et al. (45) did not find a significant age–well-being interaction in the case of short-term immune functioning, endocrine response, longevity, and symptom control.

Our study has some limitations. First, disease incidence was assessed using a self-report measure; the validity of self-report measures varies according to disease outcome. Studies have reported high agreement between self-report and clinically derived diagnosis in the case of cardiovascular diseases, diabetes (46), and cancer (47); lower levels of agreement have been found for osteoarthritis (46) and respiratory diseases (48), although others have demonstrated that self-report measures provide a valid estimate of disease prevalence for arthritis (49) and chronic lung disease (50). Fatal disease incidence—particularly relevant in the case of heart disease and stroke—was not recorded and therefore could not be included in the analysis. Second, the effect of potential bias due to missing data cannot be ruled out as participants with missing CASP-19 data were more likely to be diagnosed as having chronic lung disease and less likely to be diagnosed as having cancer. Third, the association between well-being and chronic lung disease was not significant after excluding incident cases within the first 2 years of follow-up suggesting an effect of reverse causation. However, the lower statistical power in this analysis (due to the inclusion of fewer incident cases) could also account for this result. Fourth, the CASP-19 has been criticized for the inclusion of two items that refer specifically to health- or to age-related problems that might be interpreted by the respondent to include health (51). After removal of these two items from the CASP-19 score, estimates for the associations between well-being and incident arthritis or chronic lung disease were very similar to those obtained with the original score, whereas that with diabetes incidence was slightly weaker. This suggests that the inclusion of these two health-related items in the CASP-19 is not having an undue influence on our findings. Fifth, we were unable to differentiate between different forms of cancer; different forms of cancer vary significantly with regard to etiology and time course and thus may be differentially related to prior well-being. Finally, a number of potentially relevant covariates were not included in the analysis, namely, diet, sleep quality, perceived stress, and anxiety. Strengths of the study should also be noted. We were able to adjust for potential confounders (including depression) and several potentially important mediators. Additional strengths included separate analysis across a range of disease outcomes and the fact that the sample is representative of people older than 50 years living in England (19).

To conclude, this study provides further evidence for the health-protective effect of well-being in the case of disease incidence. In addition to corroborating previous results regarding the association between well-being and incidence of stroke, diabetes, and myocardial infarction, the study demonstrates that this association extends to other chronic conditions including arthritis and chronic lung disease. The study illustrates how the association between well-being and disease incidence varies according to the disease outcome. Finally, the results provide further insight regarding the mechanisms underlying the association between well-being and disease incidence. Factors including health behaviors, BMI, depression, and demographic variables seem to account for the association between well-being and incident myocardial infarction and stroke. Additional factors may be implicated in the associations between well-being and arthritis, diabetes, and chronic lung disease.

## Supplementary Material

SUPPLEMENTARY MATERIAL
