# Supplementary material for: Well-Being and Chronic Disease Incidence: The English Longitudinal Study of Ageing
Source: Psychosom Med. 2016 Apr 11;78(3):335–44. doi: 10.1097/PSY.0000000000000279 (PMC4819773; doi:10.1097/PSY.0000000000000279)
Supplement: SUPPLEMENTARY MATERIAL [file psm-78-335-s001.docx]

| **Table S1.**  **Hazard ratios (95% confidence intervals) from analysis with wave 1 covariates and from analysis with time dependent covariates for incident arthritis, cancer, stroke, diabetes, heart attack and chronic lung disease according to a SD increase in CASP-19 score** | | | |
| --- | --- | --- | --- |
| **Chronic**  **Disease** | **Analysis** | **Model 1**  HR(95%-CI) | **Model 2**  HR(95%-CI) |
| Arthritis | Wave 1 covariates | 0.84 (0.78-0.89)** | 0.89 (0.83-0.96)* |
|  | Time dependent covariates | 0.82 (0.76-0.89)* | 0.90 (0.82-0.99)* |
| Cancer | Wave 1 covariates | 1.01 (0.91-1.13) | 1.05 (0.92-1.20) |
|  | Time dependent covariates | 1.02 (0 .90-1.15) | 1.05 (0.91-1.22) |
| Stroke | Wave 1 covariates | 0.84 (0.75-0.95)* | 0.94 (0.81-1.09) |
|  | Time dependent covariates | 0.84 (0.72-0.99)* | 0.89 (0.73-1.09) |
| Diabetes < 65 | Wave 1 covariates | 0.68 (0.61-0.75)** | 0.82 (0.71-0.95)* |
|  | Time dependent covariates | 0.65 (0.57-0.74)** | 0.81 (0.68-0.96)* |
| Diabetes ≥ 65 | Wave 1 covariates | 0.92 (0.89-1.05) | 1.01 (0.85-1.20) |
|  | Time dependent covariates | 0.91 (0.77-1.07) | 1.09 (0.88-1.34) |
| Heart attack < 65 | Wave 1 covariates | 0.71 (0.58-0.87)* | 0.84 (0.63-1.12) |
|  | Time dependent covariates | 0.71 (0.54-0.95)* | 0.78 (0.54-1.15) |
| Heart attack ≥ 65 | Wave 1 covariates | 0.79 (0.66-0.93)* | 0.86 (0.69-1.06) |
|  | Time dependent covariates | 0.82 (0.66-1.03) | 0.97 (0.73-1.28) |
| Lung disease <65 | Wave 1 covariates | 0.58 (0.51-0.67)** | 0.80 (0.66-0.97)* |
|  | Time dependent covariates | 0.60 (0.51-0.71)** | 0.79 (0.63-0.98)* |
| Lung disease ≥ 65 | Wave 1 covariates | 0.73 (0.62-0.86)** | 0.87 (0.71-1.06) |
|  | Time dependent covariates | 0.69 (0.56-0.86)* | 0.85 (0.65-1.11) |
| ^a^ Cox proportional hazards regression, estimates for diabetes, heart attack and chronic lung disease are stratified by age.  **Model 1:** Adjusted for age and sex. **Model 2:** Further adjusted for total net wealth education and relationship status, history of hypertension for diabetes and history of diabetes and hypertension for heart attack and stroke, CESD score, smoking, alcohol intake, physical activity and BMI.  ** *p* <0.001 * *p* <0.05 | | | |
